# Enhancing acute stroke care in Ireland: A scoping review and Delphi consensus for the Irish National Audit of Stroke (INAS) dataset

**DOI:** 10.1136/bmjopen-2025-102789

**Published:** 2025-11-21

**Authors:** Catherine Nora Moran, Isabelle Jeffares, Joan McCormack, Niamh A Merriman, Carlos Bruen, Agnes Jonsson, Paul Murphy, Khyber Afridi Rabbi, Joseph Harbison, David Williams, Peter Kelly, Rónán Collins, Eithne Sexton, Frances Horgan, Máirín Ní Bheacáin, Elaine Byrne, John Thornton, Collette Tully, Anne Hickey

**Affiliations:** 1 School of Population Health, RCSI, Dublin, Leinster, Ireland; 2 National Office of Clinical Audit, County Dublin, Ireland; 3 School of Public Health, Physiotherapy, and Sports Science, University College Dublin, Dublin, Leinster, Ireland; 4 Centre for Health Policy & Management, Trinity College Dublin, Dublin, Ireland; 5 School of Population Health & School of Medicine, RCSI, Dublin, Leinster, Ireland; 6 Library Services, RCSI, Dublin, Leinster, Ireland; 7 Department Geriatric and Stroke Medicine, St James Hospital, Dublin, Ireland; 8 School of Medicine, Trinity College Dublin, Dublin, Leinster, Ireland; 9 Department of Geriatric and Stroke Medicine, RCSI, Dublin, Leinster, Ireland; 10 Department of Geriatric and Stroke Medicine, Beaumont Hospital, Dublin, Ireland; 11 Department of Neurology, Mater Misericordiae University Hospital, Dublin, Ireland; 12 Neuromuscular Clinical Science Unit, University College Dublin, Dublin, Ireland; 13 Department of Geriatric and Stroke Medicine, Tallaght University Hospital, Dublin, Ireland; 14 School of Physiotherapy, RCSI, Dublin, Leinster, Ireland; 15 Patient Representative, Dublin, Ireland; 16 Centre for Positive Health Sciences, RCSI, Dublin, Leinster, Ireland; 17 Department of Radiology, Beaumont Hospital, Dublin, Ireland

**Keywords:** Stroke, Quality Improvement, AUDIT, Delphi Technique, REGISTRIES

## Abstract

**Abstract:**

**Objectives:**

To develop an updated core dataset for acute stroke care in Ireland, informed by international audit benchmarking and national stakeholder consensus, for integration into the Irish National Audit of Stroke (INAS).

**Design:**

Scoping review and three-round Delphi process.

**Data sources:**

Medline Ovid, Embase, CINAHL EBSCOhost, Google Scholar, audit websites and grey literature (2010–2024). Additional audit documentation was obtained via direct author contact.

**Eligibility criteria for selecting studies:**

National stroke audits or registries with a country-wide scope, ≥1 year of continuous data collection and active in 2021 were eligible. Only audits covering acute stroke care were included in this study phase. All records were screened for inclusion.

**Data extraction and synthesis:**

Audit documentation (data dictionaries, item definitions and contextual metadata) was retrieved from eligible audits. Acute stroke care items were extracted, charted and benchmarked against existing INAS items and each other to identify commonalities and gaps. Frequently collected international items (appearing in ≥4 audits/registries) were shortlisted. A three-round Delphi process with 24 national stakeholders (clinicians, nurses, allied health professionals, researchers, policymakers and patient representatives) was conducted to audit and refine the dataset through structured, anonymised item rating, iterative feedback and consensus-building discussions.

**Results:**

Twenty-one eligible international stroke audits/registries were identified, yielding ~4500 audit items. Benchmarking against existing INAS items (n=103), frequently collected international items (n=97) and expert-suggested items (n=22) informed the Delphi consultation. The final dataset expanded INAS by 18 items, totalling 86 acute care and 35 thrombectomy-specific items. New additions included stroke-related complications and risk factor documentation.

**Conclusions:**

This structured, consensus-led process resulted in an internationally benchmarked, stakeholder-informed core dataset to enhance standardised stroke auditing in Ireland. The expanded dataset supports enhanced clinical monitoring, quality improvement and health system planning. This approach may inform audit development and research efforts in other contexts.

**STRENGTHS AND LIMITATIONS OF THIS STUDY**

- A comprehensive international scoping review was conducted using a systematic and reproducible search strategy.
- Non-English audit documentation was translated and verified to enhance data accuracy and inclusivity.
- A three-round Delphi consultation followed best practices for consensus-building, including pseudonymous voting and iterative feedback.
- Variability in audit structures, definitions and data collection methods posed challenges for direct international comparisons.
- Reliance on existing audit documentation may have excluded emerging or unrecorded aspects of stroke care.

## Introduction

 Stroke is the second most common single cause of mortality in the European Union ^1^ and the predominant reason for acute hospitalisations in high-income countries.^2^,^4^ It is a leading contributor to adult-acquired disability,^4 5^ resulting in a broad spectrum of post-stroke sequelae from short-term to persistent functional, communication, psychological and cognitive deficits.^6^,^9^

Variability exists in stroke outcomes across hospitals and geographic locations,^10^,^13^ highlighting the need for ongoing and continual review of stroke healthcare delivery to ensure high-quality and equitable best practice is being delivered. By prospectively and continuously tracking consecutive stroke admissions, national clinical audit makes it possible to record the proportion of patients receiving care that meets clinical standards and discern any variation in quality or access across hospitals, regions or time periods. While the terms audit and registry are often used interchangeably, a registry primarily refers to the structured, longitudinal collection of patient-level clinical data, whereas an audit encompasses the systematic process of using that data to assess compliance with predefined standards, support benchmarking and drive quality improvement.^14 15^ In practice, this distinction often overlaps, particularly when national datasets serve both data collection and quality improvement functions.

Stroke audits allow for the identification of specific care processes and treatments that are associated with better patient outcomes.^16 17^ Audit data at a national level can be used to monitor trends and benchmark against international standards, enabling healthcare decision-makers at local, regional and national levels to institute policy change, identify care priorities and address service delivery gaps. Countries with hospital-based national clinical audits or registries of stroke care with routine data collection have observed the availability of stroke audit data contributing to improvements in health policy, stroke care delivery and, as a result, to patient outcomes.^18 19^

In Ireland, the first Irish National Audit of Stroke Care^20^ revealed significant shortcomings in the provision of stroke services across the hyperacute, acute and rehabilitation phases of care.^20^ In response, the National Stroke Programme (NSP) was established in 2010, leading to the development of a National Stroke Register to systematically collect data on stroke care delivered in public hospitals to target quality improvement initiatives.^21^ In 2019, governance of the Register was assumed by the National Office of Clinical Audit, at which point the NSP was formalised and renamed as the Irish National Audit of Stroke (INAS).^22^ INAS is a clinically led, continuous national clinical audit, overseen by a multidisciplinary Governance Committee comprising clinical experts, researchers, senior health system administrators, and public and patient representatives. This transition initiated a focus on updating the core dataset through a process of international benchmarking and stakeholder consensus.

INAS collects data via a web-based portal from hospital sites across Ireland on in-patients with acute stroke. Key performance indicators are measured against Irish^23 24^ and United Kingdom (UK)^25^ guidelines and joint UK-Ireland stroke care guidelines.^26^ The audit sources demographic, clinical and administrative data from an existing national database of discharges from acute public hospitals in Ireland (Hospital In-Patient Enquiry (HIPE) system). It collects stroke-specific clinical data on all patients who have experienced a stroke (‘Core Clinical’ dataset), submitted by hospitals to HIPE via a stroke audit portal. Additionally, thrombectomy data (‘Thrombectomy’ dataset) are collected on patients who receive a thrombectomy from one of the two endovascular thrombectomy (EVT) centres in Ireland. Since 2018, separate rehabilitation and recovery data are collected by health and social care professionals (HSCPs) in participating hospitals (‘HSCP’ dataset). To participate in the acute audit of the INAS, hospitals are required to meet inclusion criteria, including ≥80% data coverage and the admission of at least 25 stroke patients annually. According to the most recent national report, overall hospital participation was 90%.^10^ The audit has implemented rigorous quality assurance measures, including quarterly data validation reports, to address data gaps and anomalies. Governance structures in participating hospitals, which include clinical leads and audit coordinators, are central to ensuring high-quality data submission and audit participation. INAS provides a robust framework for monitoring stroke care, fostering alignment with evidence-based guidelines and driving quality improvement across Ireland.^27^

Despite the availability of stroke audits and registers internationally, a number of challenges prevail. Many countries do not have a national stroke registry or audit, with only 14% of WHO member countries and territories having what is described as optimal stroke surveillance through a national stroke registry.^28^ Where such national registries or audits are available, there are frequently limitations that pose challenges to data interpretation and application. These include lack of consistency in definitions, in the methods used to gather data, errors in data entry with no data validation checks built into the data collection process, lack of precision in coding of stroke, and lack of international benchmarking^27^,^29^

The aim of this research was to develop INAS by establishing a core minimum dataset for acute care (Phase 1), starting with a scoping review of international stroke audit, followed by an international benchmarking exercise of stroke audit items, and iterative cycles of expert stakeholder engagement to agree a final acute audit dataset. The development of the audit dataset for rehabilitation and recovery care (Phase 2) and the identification of resourcing needs and production of an implementation strategy (Phase 3) are reported separately.^27^ This approach of combining a scoping review with a Delphi process enabled us to ensure that the process was well-informed and evidence-based, enabling stakeholders involved in the Delphi process to make decisions about the items suitable for inclusion in and exclusion from INAS based on best current evidence of international stroke audit.

## Materials and methods

### Protocol and registration

The protocol for this scoping review was published^30^ and registered with Open Science Framework (OSF Registries).^31^ The scoping review was conducted in accordance with the six-stage stepwise methodological framework specified by Arksey and O’Malley and advanced by Levac *et al* and Peters *et al*.^32^,^34^ The scoping review findings are reported in line with the Joanna Briggs Institute (JBI) Preferred Reporting Items for Systematic Reviews and Meta-Analysis Extension for Scoping Reviews (PRISMA-ScR) reporting guidelines.^35^ This study is also reported in accordance with the ACCORD (ACcurate COnsensus Reporting Document) Guideline.^36^

No formal ethics approval was required for this study as it was a scoping review of existing literature, which does not involve human participants. The Delphi consensus process, used to refine the outcomes from the scoping review, was considered an audit activity, in line with established definitions of audit (https://hseresearch.ie/what-is-research-2/), and therefore did not require Research Ethics Committee (REC) approval. Participants were fully informed about the process, and their involvement was voluntary, as evidenced by their completion of tasks via email links. Participation was initially pseudonymised to allow for data linkage across the audit rounds and was subsequently irrevocably anonymised.

### Eligibility criteria

Peer-reviewed and grey literature were systematically searched for national and continuous international stroke audits and registries published since 2010. We limited inclusion to sources from 2010 onward to ensure the data reflected contemporary clinical practice and actively maintained audits relevant to current benchmarking. In line with the recommended scoping review PCC (population, concept, context) approach, our inclusion criteria comprised:

- **Population:** Stroke (any type).
- **Concept:** National stroke audit, defined as a data collection programme (register, databank or database) used for measuring and monitoring the quality and structure of stroke care services and performance indicators across multiple participating sites for patients hospitalised with stroke.^14^ We acknowledge the existence of numerous state-wide and regional stroke audits, for example, the Registry of Stroke Care Quality (RES-Q),^37^ among others. However, in delineating the scope of the review and to manage the volume of potential audit items for benchmarking, we chose to focus on national-level audits for comparison, aligning with the national scope of the Irish audit.
- **Context:** The stroke audit or registry operated as the established national country-wide system for stroke care data collection, bore the country’s name (as guided by the names of the United Nations member states, or constituent country of a member state, and including Taiwan), or included the term ‘national’ within its title. Audits pertaining to care quality across both acute and non-acute stroke care settings were considered for inclusion. Only audits and registries with continuous data collection, that had at least 1 year of prospective data collection and were still functioning in 2021, were included.

### Information sources

Electronic databases (Medline Ovid, Embase and CINAHL EBSCOhost) were searched for peer-reviewed literature published in English since 2010 to identify national stroke audits or registries. A targeted search for relevant grey literature was also performed by searching Google Scholar and websites of interest, such as stroke organisation websites and reports.^38 39^ A broad range of published and unpublished evidence sources was eligible, including primary research studies, systematic reviews, meta-analyses, website reports, guidelines and recommendations. Conference abstracts were excluded. This search strategy was developed in consultation with an information specialist (PM). Peer-reviewed literature searches were limited to English-language publications. However, for audits meeting inclusion criteria, supplementary audit documentation (eg, data dictionaries, methodology reports) obtained via audit/registry websites or through direct correspondence with audit/registry leads was included regardless of language and translated to English where necessary to enable data benchmarking.

### Search strategy and selection criteria

Identified databases were searched from 2010 using medical subject headings (MeSH) and text words on 10 February 2021, and updated on 4 March 2024. Search terms included “stroke”, “transient ischaemic attack”, “intracerebral haemorrhage”, “national stroke registry”, “stroke register”, and “stroke audit” (online supplemental file 1). All results were imported into EndNote X9.3.3 and duplicates removed. To ensure literature saturation, a snowball approach was adopted; as sources of evidence were identified, the reference lists (ascendency search) and papers that cited the sources (descendency search) were scanned for relevant materials. Once an eligible audit was identified, a more specific search approach was employed to obtain information regarding audit datasets, data dictionaries and data collection procedures. Where the documentation was not published or available on the audit website, authors/audit managers were contacted. Audit documents that were provided in a language other than English were translated and included in the data charting and synthesis stages. Non-English audit documents were translated into English using a professional certified translation service (Certified Translations, Dublin, Ireland (*www.certifiedtranslations.ie*)) to ensure data accuracy and completeness during synthesis.

### Selection of sources of evidence

Two independent reviewers from a bank of reviewers (CB, NAM, KAR, AH, and GH (see Acknowledgements)) initially screened titles and abstracts against predefined inclusion and exclusion criteria.^30^ Following this, the full texts for potentially relevant sources were retrieved and further assessed for eligibility. At each stage, discrepancies were resolved through consensus-based discussion and consultation with a third reviewer (AH), when necessary. INAS was considered within the scope of the review and was included in the charting and synthesis stages.

### Data charting process

The previous scoping review stages allowed for the inclusion of sources of evidence relating to national stroke audits of all types. Audits that encompassed both acute and non-acute stroke care were eligible, provided they included a continuous and structured acute care component. During the data charting and synthesis steps, the included audit documents were categorised as relating to acute stroke care (Phase 1) or non-acute stroke care (Phase 2; this phase is reported separately). For the phase of the review reported here, only data items related to acute stroke care were extracted and analysed.

Data relating to stroke care audits were extracted by two independent researchers (CNM, NAM) from the retrieved documentation and charted into a Microsoft Excel proforma. A data charting calibration check was conducted whereby data extracted by both reviewers for a random sample of one-third of the databases were compared. The level of inter-rater agreement was 95%, and any discrepancies (eg, missing data or uncertainty) were resolved through consensus-based discussion. As agreement was high, data charting for the remaining audit documents was conducted by either one of the researchers. The results of the data charting exercise were discussed with the INAS Governance Committee (a group of national experts established to provide ongoing oversight of INAS) in an iterative process that facilitated, where appropriate, the potential revision of the charting framework to ensure that data were captured comprehensively. Any missing details were sought through additional searching or further contact with the relevant audit report authors.

### Data items

The following stroke care items were extracted from the international stroke audit documentation: (a) audit characteristics and context (setting, phase of care covered, eligible patient population, number of sites, follow-up) and (b) acute audit data items (audit question/item, response options).

### Critical appraisal of individual sources of evidence

The quality of the individual sources of evidence was not formally assessed as the aim of a scoping review is to provide an overview of the breadth and current status of available evidence on a topic from different source types and not to assess the weight or robustness of the evidence.^32 34^

### Synthesis of results

#### International benchmarking of acute stroke care items

Once the data items were collated, a two-step benchmarking exercise was conducted whereby the acute stroke care items present across the retrieved international audit databases were cross-checked to identify commonalities and/or gaps in coverage. In the *first* step, we identified which items the Irish stroke audit shared with international stroke audits and registries. With INAS as the key comparator, each of the Irish Core Clinical and Thrombectomy items was cross-checked against all other included international audits and registries to look for equivalent or closely related acute stroke care items. Frequency counts, representing the number of audits that contained the same (or closely related) item, were tallied for each INAS item to show which Irish items are commonly or infrequently collected internationally, or perhaps are unique to the Irish healthcare context. An INAS item was classified as low shared if present in 0–2 other audits/registries, moderately shared if present in 3–10, and highly shared if present in 11–20.

In the *second* step, we aimed to identify which other acute items were collected internationally (and how frequently) that were not captured by INAS. With the Australian registry (Australian Stroke Clinical Registry (AuSCR)^40^) as the key comparator (chosen as it represented the most comprehensive stroke registry), any additional acute stroke care items were cross-checked to see how frequently other international audits and registries asked something equivalent or closely related. Following this primary benchmarking, a review of all remaining international audits was conducted to identify any additional acute care items not present in either INAS or AuSCR. These items were also cross-checked across the full audit set to determine their frequency of use. For transparency, the country of the audit where each item was first identified is noted as its source of origin. Frequency counts were generated to display how many international audits collected each acute data item that the Irish audit does not currently collect. An international reference item not collected by INAS was classified as moderately shared if present in 3–10 other audits/registries, and highly shared if present in 11–19.

The benchmarking process generated an inventory of acute stroke care items currently in use (Irish audit and international items) with frequency mapping detailing how common or uncommon each item is in international practice. Following consultation with the project Steering Group, a criterion was set such that only predominant frequently collected international items, that is, those items shared among four or more audits/registries in total (ie, the comparator audit item and at least three other audits/registries), were put forward for further consideration by a stakeholder panel.

#### Stakeholder Delphi consultation on the acute stroke care dataset

A Delphi panel was established through a collaborative process involving the INAS Governance Committee and project Steering Group. Stakeholders with expertise in stroke care were invited to participate in a multi-round Delphi consultation aimed at evaluating and refining existing INAS items, as well as frequently-collected international items (used in four or more national audits or registries). Participants received a pseudonymised, Excel-based voting form listing all audit items under consideration.

The panel included 24 members representing a diverse range of national expertise in stroke: senior stroke clinicians (n=8), stroke specialist nurses (n=1 stroke advanced nurse practitioner, n=1 stroke clinical nurse manager, n=1 director of nursing), stroke and health services researchers (n=4), patient representatives (n=2), professionals in national stroke policy, patient safety and service planning (n=5), and senior allied healthcare professionals specialising in stroke care (n=1 physiotherapy, n=1 psychology). One physiotherapist held an academic appointment and was therefore included under the researcher category. A majority of stakeholders (n=18) were very familiar with INAS and almost all healthcare professional stakeholders were involved in inputting or overseeing the input of data from their hospital to the INAS database. Stakeholders included the National Lead of the Stroke Programme in Ireland, the Clinical Lead of INAS and the INAS Programme Manager. This inclusive selection ensured broad representation of relevant national stakeholders, particularly those who oversee INAS audit content and implementation, to enhance the validity and applicability of the audit’s findings. Participant identities were initially pseudonymised to enable linkage of responses across Delphi rounds and were subsequently irreversibly anonymised prior to final data analysis. The Delphi consultation took place between March and December 2022, with each voting round allowing a 2 week response period.

Across three rounds of Delphi consultation, stakeholders engaged in a structured voting process, providing expert opinions on all of the existing Irish audit items, independent of benchmarking frequencies, and evaluated whether the current INAS Core Clinical and Thrombectomy items should be retained in, or omitted from, INAS. The panel were also asked to audit the proposed inventory of frequently collected international items and to consider whether any of the items should be added to INAS as part of the core minimum acute stroke care dataset. Throughout the Delphi rounds, stakeholders were instructed to assess items based on their importance for Irish national audit purposes, independent of current resource constraints.

Each item was rated as “Include in INAS”, “Exclude from INAS”, “No strong view” or “Don’t know enough to be able to say”, with space provided for brief optional qualitative comments. These comments were anonymised and summarised by the research team to contextualise voting patterns and inform discussion. Summarised feedback was presented during Steering Group and Governance Committee meetings between voting rounds, and meeting transcripts containing aggregated, anonymised comments were circulated to all participants. This process promoted transparency, allowed participants to reflect on group perspectives and informed subsequent voting. In Round 2 of the Delphi process, participants had the opportunity to revise their initial responses in light of this comparative feedback and group-level voting patterns. Although qualitative comments were not formally coded, they were instrumental in guiding iterative consensus-building throughout the process. Based on stakeholder engagement, additional items were proposed during this stage and incorporated into the inventory for consideration in the final Delphi round.

In the final round of the Delphi process (Round 3), participants voted on a reduced set of items based on the outcome of the first two rounds, as well as the additional expert-suggested items, using simplified response options: “Include in INAS”, “Exclude from INAS” or “Don’t know enough to be able to say”. Anonymised summaries of prior discussions were provided to guide decisions. Final consensus levels were reviewed by the Steering Group and Governance Committee, which provided clinical and policy justification for the resulting core minimum dataset.

Delphi voting thresholds and procedures were established a priori. Items were classified according to consensus thresholds based on the mean percentage of votes for inclusion in INAS: high consensus (≥70%), moderate consensus (50–69%), and low consensus (<50%), applied separately to Irish and international acute stroke care items. Items with ≥70% support for inclusion were considered for the final INAS acute outcome dataset. Detailed voting instructions for each Delphi round are available in online supplemental file 2.

### Patient and public involvement

Patient and public involvement (PPI) has been central to this research, with stroke survivors contributing at every stage. A stroke survivor was part of the coapplicant team during the research grant application, ensuring the patient perspective was included from the start. The development of INAS has been a collaborative process, involving ongoing engagement between the research team, collaborators, knowledge users and patient representatives. Two stroke survivors provided feedback on the scoping review, participated in the Delphi panel and highlighted key aspects of stroke care to prioritise in the audit. Additionally, the Head of Advocacy at the Irish Heart Foundation (IHF) was a collaborator on the project and has ensured patient and family member perspectives are integrated throughout, using the expertise of the advocacy team to keep the research aligned with stroke survivor and family member’s priorities. This iterative PPI approach has shaped the study’s objectives, ensuring the voices of stroke survivors are central to the design and conduct of the research.

## Results

### Selection of sources of evidence

The initial scoping review search yielded 5396 records, of which 263 duplicates were removed. Two independent reviewers screened 5133 titles and abstracts for eligibility. Subsequently, 871 full texts were reviewed, from which 27 national stroke audits and registries were identified, including the Irish stroke audit. As the unit of inclusion was the audit or registry rather than individual articles, multiple sources could refer to the same audit or registry. After further review, four of the identified stroke audits/registries were excluded owing to non-continuous data collection at the time of the search, namely, the Chinese National Stroke Registry,^41^ the Finnish National Stroke Database,^42^ the Malaysian National Neurology Registry^43^ and the Thai Stroke Registry.^44^ An additional two-stroke registries were excluded as we were unable to obtain the relevant audit documentation or information detailing the data items, despite numerous requests (the Polish Stroke Registry^45 46^ and the Russian National Stroke Registry^47^). Included audits comprised the Australian stroke registry (AuSCR),^40^ the Austrian Stroke Unit Registry,^48^ CorHealth Ontario (Canada),^49^ DanStroke (Denmark),^50 51^ the German Stroke Register Study Group (ADSR),^52^,^54^ the Irish National Audit of Stroke (INAS),^22^ the Japan Stroke Data Bank,^55^,^57^ the Dutch Acute Stroke Audit (DASA),^58^ the Norwegian Stroke Registry (NHR),^59 60^ the Scottish Stroke Care Audit (SSCA),^61^ the Singapore Stroke Registry,^62^ the Slovak Stroke Register,^63^ the Clinical Research Collaboration for Stroke in South Korea (CRCS-K),^64^ the Korean Stroke Registry (KSR; South Korea),^65^ the Spanish national stroke audit (established under the Spanish Society of Neurology (SEN); RENISEN),^66^ the Swedish national stroke register (Riksstroke),^18 67^ the Swiss Stroke Registry,^68 69^ the Taiwan Stroke Registry,^70^ the Sentinel Stroke National Audit Programme (SSNAP; UK),^71 72^ Get with the Guidelines Stroke Registry (GWTG; USA),^73^ and the Paul Coverdell National Acute Stroke Programme (PCNASP; USA).^74^ Audit documentation for five of the included stroke registries was available in languages other than English and was translated.^48 51 54 57 59^

**Figure 1:**
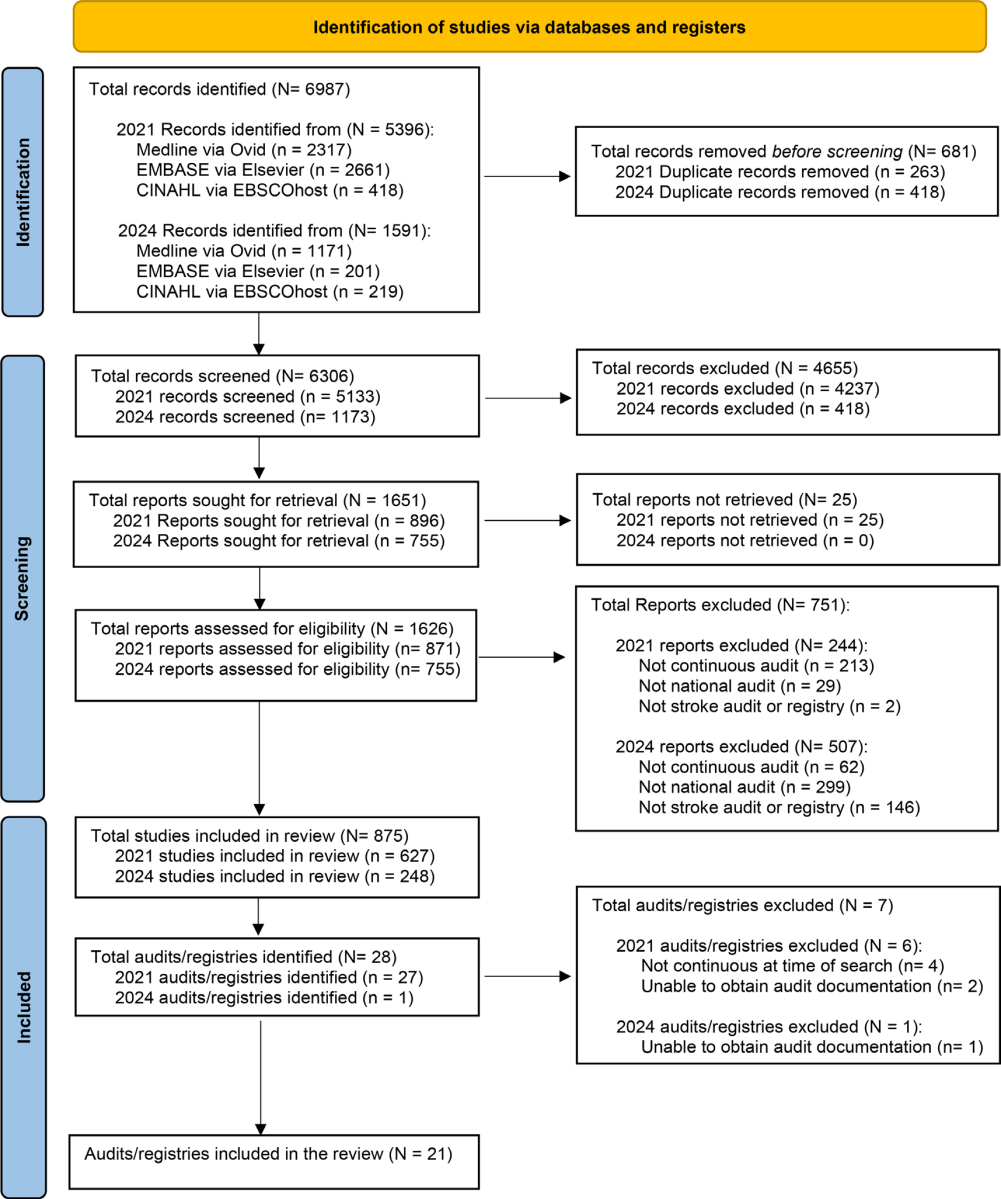
Preferred Reporting Items for Systematic Reviews and Meta-Analyses (PRISMA) flow diagram detailing the scoping review search strategy, the number of identified citations screened, reviewed and excluded, and number of audits included. Note. Inclusion was at the audit/registry level; multiple sources could inform a single audit, so article counts are not reported.

The database search was updated in March 2024, resulting in 1591 additional titles since the initial search. Following the removal of 418 duplicates, 1173 titles and abstracts were screened for eligibility. Following exclusions, 755 records were retrieved for review. Only one additional national stroke audit/registry was identified: the Israeli National Stroke Registry.^75 76^ During the initial search, the Israeli National Stroke Registry may have been conflated with the National Acute Stroke Israeli Survey, which is conducted triennially and is therefore non-continuous, and as a result was excluded. After numerous requests, we were unable to obtain the necessary registry documentation, leading to its exclusion from the review. As follows, twenty-one stroke audits/registries (across 19 countries) with national coverage and continuous data collection were included in the scoping review (see figure 1).

### Characteristics of sources of evidence

This review incorporates 21 national and continuous audits and registries, the characteristics of which are detailed in online supplemental file 3. Most of the audits and registries initiated data collection in the late 1990s and early-to-mid 2000s and all remain operational. All audits and registries collected data on patients with ischaemic and haemorrhagic stroke, and two-thirds of registries also included patients with a transient ischaemic attack (TIA). Audits and registries varied with respect to whether there was voluntary involvement by participating hospitals or whether data acquisition was government mandated.

### Results of individual sources of evidence

#### Results of the international benchmarking of acute stroke care items

Following data extraction, an inventory of the acute stroke care data items from 21 included stroke audits and registries was compiled. This inventory comprised almost 4500 items derived from 20 international stroke audits/registries, and a further 103 items from the Irish stroke audit. Specifically, the Irish audit included 10 items from the HIPE system, 58 items comprising the ‘Core Clinical’ dataset, and 35 items comprising the ‘Thrombectomy’ dataset. Comparison of INAS items with the international inventory showed varying levels of overlap: of the 103 INAS items, 40 were classified as highly shared, 48 as moderately shared, and 15 as low shared or not collected elsewhere (see online supplemental file 4 for full frequency data). This benchmarking exercise highlighted both areas of alignment with international practice and opportunities for dataset enhancement.

Analysis of the international audit inventory identified 97 acute stroke care items not currently included in INAS but frequently collected elsewhere (these items appeared in at least four international stroke audits or registries (see online supplemental file 5)). Of these, 90 were used by a moderate number of audits, while seven were commonly collected across a high number of registries. This subset of frequently collected international items was brought forward for stakeholder review to inform potential expansion of the INAS dataset.

### Synthesis of results

#### Results of the stakeholder Delphi consultation

*Delphi Round 1:* Twenty out of 24 eligible experts in stroke responded to Round 1 of the Delphi exercise (83% response rate) involving audit of the proposed inventory of items. The group voting averages for each response option pertaining to the INAS and international audit items are presented in online supplemental files 6,7, respectively. After Round 1, 56/103 INAS items received a high percentage of votes (≥70%) for inclusion (in this case, retention) as part of INAS (online supplemental file 6). Of the 97 frequently collected international items, 17 items obtained high votes (≥70%) for inclusion in (or in this case, addition to) INAS (online supplemental file 7).

*Delphi Round 2:* In Round 2 of the consultation, the initial group of responding stakeholders (n=20) reviewed their previous responses alongside the mean aggregated group responses for each audit item under consideration. One stakeholder had left their position and did not engage beyond Round 1 of the process, but their responses were retained in the Round 2 dataset. Of the remaining 19, eleven stakeholders responded; nine submitted revised responses for one or more items, while two confirmed their original votes. The eight non-responding stakeholders were assumed to endorse their initial responses.

Across all stakeholder responses, a total of 66 INAS item responses (3.2% of all possible INAS responses, based on 103 items) and 42 international item responses (2.2% of all possible international responses, based on 97 items) were revised between Rounds 1 and 2 (see online supplemental file 8 for details). Of the 66 INAS item changes, 44 (66.7%) shifted towards “Include”, 15 (22.7%) towards “Exclude” and 7 (10.6%) towards “No strong view.” For the 42 international item changes, 10 (23.8%) shifted towards “Include,” 17 (40.5%) towards “Exclude,” 5 (11.9%) towards “Don’t know” and 10 (23.8%) towards “No strong view.” Vote distributions in online supplemental files 6,7 represent combined responses from all 20 stakeholders, including unchanged votes from eight non-responders and two who confirmed their initial responses, along with updates from nine participants who submitted revisions.

Following Round 2, 61 INAS items (ie, 10/10 HIPE, 44/58 Core Clinical and 7/35 Thrombectomy items) garnered a high percentage of votes (≥70%) for inclusion (retention) in INAS (online supplemental file 6). Eleven Core Clinical items and 20 Thrombectomy items received a moderate percentage of votes (50–69%) and 3 Core Clinical and 8 Thrombectomy items received a low proportion of votes (<50%) for inclusion. Among the 97 international items, 24 items obtained a high percentage of votes for inclusion as part of INAS, 25 received moderate votes and 48 received a low proportion of votes (online supplemental file 7).

Based on these results, the low-voted INAS Core Clinical (n=3) and low-voted international (n=48) items were excluded from further consideration. The highly-voted Irish items (n=61) were recommended for ongoing retention in INAS without the need for further deliberation. An additional 22 items, derived from stakeholder input during Rounds 1 and 2, were subsequently added to the inventory of audit items for consideration in Round 3, alongside the remaining items that required consensus (ie, the INAS Core Clinical and international items with moderate endorsement, and the highly-voted international items).

*Delphi Round 3:* In the third round, 17/22 eligible stakeholders (77% response rate) voted on, and discussed in a subsequent stakeholder meeting, the remaining inventory of items to refine and finalise their potential inclusion in the recommended prioritised core acute dataset. Two INAS Core Clinical items received a high percentage of votes for inclusion and hence were recommended for ongoing retention in INAS (online supplemental file 6). Six Core Clinical items received a moderate proportion of votes for inclusion, while three Core Clinical items received a low proportion of votes which did not meet the inclusion cut-off and were suggested for exclusion from the final dataset. Regarding the INAS Thrombectomy items, following consultation with the stakeholders, a decision was made that the Thrombectomy dataset should be retained in its entirety in the short term for the two EVT centres to which it applies to support the current ongoing development of a National Thrombectomy Service in Ireland.

For the remaining items under consideration, 24 international items and 6/22 additional expert-suggested items were highly voted and thus were proposed for inclusion in INAS (online supplemental file 7). Following the stakeholder consultation, 16 moderately voted and 9 low-voted international items, along with 10 moderately voted and 6 low-voted additional expert-suggested items, were excluded from INAS.

*Final recommended core minimum dataset for acute stroke care in Ireland:* Following the international benchmarking and multiround stakeholder consultation processes, a consensus was achieved on the final recommended core minimum dataset for acute stroke care in Ireland, totalling 86 items for the majority of hospitals in Ireland and 121 items for the two EVT centres (a summary is provided in table 1, with the full list of items detailed in online supplemental file 9). The final recommended dataset retains 10 items from HIPE, 46 Core Clinical items and incorporates 24 additional international items and six expert-suggested items. For the two EVT centres in Ireland, there are an additional 35 Thrombectomy items. The newly recommended additional items primarily examine history of known stroke risk factors, complications during hospital admission, details on medications before admission and at discharge, and screening for aphasia and cognitive impairment.

**Table 1:** Summary of the proposed core minimum dataset items for acute stroke care in Ireland

Original audit source	Item numbers	Content covered
HIPE	1–10	Demographics (age, sex), admission type and source, length of stay, principal and additional diagnoses
INAS Core Clinical dataset	11–56	Symptom onset/timing, imaging, thrombolysis/thrombectomy, swallow screen, stroke unit admission, allied health input, antithrombotic therapy, atrial fibrillation management, discharge destination, and mRS
INAS Thrombectomy dataset	57–91	Imaging timings, referral and transfer details, procedural steps (groin puncture, reperfusion), complications, NIHSS preprocedure and postprocedure
International audit datasets (international benchmarking)	92–115	Patient identifiers, ambulance arrival, history of vascular risk factors, preadmission medications, baseline NIHSS, in-hospital complications, discharge medications
Additional expert-suggested items (Delphi consultation)	116–121	Intracerebral haemorrhage location, mRS at 90 days, cognitive and aphasia screening, prestroke dementia diagnosis

A full list of 121 audit items is provided in online supplemental file 9.

HIPE, Hospital In-Patient Enquiry; INAS, Irish National Audit of Stroke; mRS, modified Rankin Scale; NIHSS, National Institutes of Health Stroke Scale.

## Discussion

This paper details the development of a core minimum acute stroke care dataset for integration into the INAS, designed in accordance with international best practice as identified through a scoping review of international stroke audits and refined through iterative cycles of stakeholder engagement.

We identified 24 national, continuous, hospital-based clinical stroke registries and audits, spanning 22 countries (including Ireland), that are currently in use to monitor the quality of acute stroke care. Three of these audits did not respond to repeated contact from the research team requesting their audit items^45^,^4775 76^ ; thus, 21 audits were included in this scoping review analysis. The final consensus-derived, audited and recommended core acute dataset for Ireland comprises 86 items in total for all hospitals in Ireland and an additional 35 (totalling 121 items) for the two EVT centres in Ireland. Despite numerous thrombectomy items meeting the criterion for exclusion, stakeholder discussions led to the decision to retain the Thrombectomy dataset in its entirety in the short term, to provide comprehensive data to support the ongoing development of the National Thrombectomy Service in Ireland. Regarding the 86 acute clinical items, 56 were retained from the original INAS Core Clinical dataset, while 30 new items, a combination of international items and items identified by the stakeholder panel, were recommended for inclusion to address identified gaps in data capture. Twelve items from the original dataset did not meet the cut-off for inclusion in the final recommended dataset.

The largest grouping of new items recommended for inclusion in INAS was 12 items pertaining to history of known stroke risk factors. Risk factor items are widely collected in stroke audits internationally (eg, history of ‘diabetes’, ‘previous stroke’, ‘hypertension’, ‘previous TIA*’*). Information on risk factor history is crucial for effective stroke prevention and awareness campaigns. It also plays a vital role in tracking changes in risk factor profiles as population demographics shift, facilitating the identification of vulnerable groups and the adjustment of strategies to improve survival outcomes.

Further new items recommended for inclusion in INAS concerned increasing the number of items relating to prestroke and poststroke medication uses. At present, INAS includes items that record antithrombotic therapy prior to admission for those with known atrial fibrillation, during acute treatment and at discharge (as secondary prevention). Following data synthesis, three additional medication items were recommended for inclusion in INAS, relating to antihypertensive use and lipid-lowering treatment prior to this stroke, and prescription of anti-hypertensive agents on discharge after stroke. These therapies were identified as crucial for reducing the risk of ischaemic and recurrent strokes by targeting risk factors such as hypertension^77^ and hypercholesterolaemia.^78^

Some audit items from international audits were recommended by stakeholders for inclusion in INAS not as part of continuous national data collection but as periodic and/or local audits. These items related primarily to hospital-based complications of stroke (eg, ‘aspiration pneumonia’, ‘urinary tract infection’, ‘seizures*’*), which were identified as valuable for revealing care areas needing intervention, highlighting service disparities and informing service improvements.

Stakeholders recommended the addition of two items to the Core Clinical dataset that had previously been part of the INAS HSCP dataset, relating to previous history of and screening for aphasia and cognitive impairment. Although clinical guidelines highlight the need for aphasia and cognitive screening after stroke,^26 79^ implementation of cognitive screening in particular is inconsistent.^10 79^ Nevertheless, cognitive screening is a vital first step for identifying deficits and determining immediate support needs and longer-term rehabilitation planning.^79^

Many of the items identified as priorities for INAS align with recommendations from previous efforts to standardise stroke care quality and performance audit measures, including indicators on stroke severity, acute complications, initiation of secondary prevention (eg, blood pressure management), and functional outcomes such as 90-day modified Rankin Scale (mRS).^80 81^ While most of these indicators are already within the remit of acute stroke audits, some, like the mRS at 90 days, typically fall outside the immediate hospital admission period. However, this outcome measure is commonly assessed during standard outpatient follow-up visits at 3 months post-discharge in Ireland, providing a practical opportunity for its inclusion without significant additional data burden. Consequently, the implementation of these indicators will enable systematic and comparative assessments of stroke care quality across international healthcare systems.

### Existing items recommended for exclusion or revision

Twelve existing INAS Core Clinical items were recommended for exclusion. A number were secondary items, providing free-text spaces to specify reasons not listed in a previous item’s response set. Free text was considered effortful to input, difficult to ensure data quality and challenging to analyse. The recommendation was that item response sets be as comprehensive as possible, eliminating the need for follow-up free-text items.

Other items were deemed not to be meaningful (eg, ‘case complete’) and therefore were recommended for exclusion. Finally, some items were recommended for exclusion from INAS in their current format. Specifically, three items referring to carotid stenosis were recommended for item revision. As an important risk factor for stroke, stakeholders proposed that the phrasing of international audit items relating to carotid stenosis be considered in rephrasing the Irish audit items in this domain.

### Methodological considerations

Conducting a scoping review and a Delphi consultation process with stakeholders involves several methodological considerations. The identification of national stroke registries and audits was complicated at times by ambiguous and inconsistent reporting on coverage, continuous data collection and disparities in items collected, influenced by local priorities and contextual factors as much as clinical and evidence-based considerations. Many registries, while very comprehensive and with the ambition or potential to be national audits, lacked full national coverage or a country-specific national audit framework (eg, European Stroke Organisation, ESO RES-Q,^37^ Indian stroke audit^82^ and Bigdata Observatory Platform for Stroke in China^83^) were collected modularly^41^ or had shifted from continuous or national to periodic or regional collection due to funding constraints and organisational barriers.^43 44^ Although RES-Q was excluded from our main dataset comparison, we acknowledge its broad international use and relevance to stroke quality improvement. Notably, there is substantial overlap between RES-Q items^84^ and both current and proposed INAS items. For example, RES-Q includes “mode of arrival to hospital,” which aligns with the newly proposed INAS item on whether the patient arrived by ambulance. It also captures NIHSS on arrival and a range of prestroke risk factors, which mirror several of our proposed INAS item additions. RES-Q similarly collects data on preadmission medications (eg, antihypertensives, lipid-lowering agents), as well as key post-stroke complications including pulmonary embolism (PE), urinary tract infections (UTIs) and deep vein thrombosis (DVT), all of which are now recommended for inclusion in INAS. This alignment underscores the validity of our proposed additions and suggests that future efforts to harmonise INAS with international standards may be highly beneficial, particularly in jurisdictions where RES-Q is widely used.

While such alignment with international initiatives like RES-Q is promising, broader methodological variation across stroke audits remains a challenge. The diversity in quality indicators, methodologies and data collection processes across stroke audits complicates consistent performance measurement and comparisons.^85^ To facilitate international benchmarking and performance review, data governance for national audits should ensure detailed reporting of registry data quality, item definitions, active status and coverage. We attempted to reduce selection bias by contacting authors for additional clarifications on audit eligibility and translating data dictionaries to English, where necessary.

In the Delphi consultation, we aimed to reduce potential biases such as dominant perspectives and groupthink by allowing participants ample time and opportunities to vote pseudonymously offline, review controlled feedback and change their votes. Adhering to best practices,^86 87^ we conducted three rounds of pseudonymous voting (with irrevocable anonymisation post-data collection and prior to final data analysis), held Steering Group and Governance Committee meetings to facilitate group discussion, and set a priori thresholds for consensus. The Delphi process is inherently subjective, reliant on the representativeness of the expert panel. Our expert group included a diverse range of senior stakeholders, such as stroke national clinical leads, senior stroke physicians, senior stroke nurses, audit and policy experts, patient representatives and allied healthcare practitioners, achieving good response rates across voting rounds. However, future research could benefit from a larger and more diverse panel at a national level, and also at European and international levels, to ensure that the individual national audits align with local healthcare and national priorities, as well as adhering to evolving international best practice standards and guidelines.

The scoping review and Delphi consensus process described here represents a structured approach to developing a core outcomes dataset for acute stroke care in Ireland, grounded in international benchmarking and broad stakeholder input. The findings highlight the importance of periodically reviewing audit items to maintain alignment with evolving guidelines and clinical practice, and to refine item clarity through rewording, expanded response options and improved sequencing with skip logic. Implementation of the revised dataset is now underway, led by the INAS Governance Committee in collaboration with the multistakeholder project Steering Group, with careful attention to the resourcing implications of these changes. Piloting the dataset will provide valuable insights into its feasibility and potential impact, supporting evidence-informed adjustments prior to full integration. This applied partnership approach illustrates how research can be translated rapidly into actionable audit policy and practice, ultimately strengthening the capacity for continuous improvement in stroke care.

## Conclusions

This study aimed to develop a core outcomes dataset for acute stroke care in Ireland through international benchmarking and stakeholder consensus. It provides a structured overview of global audit practices and contributes a refined, context-specific dataset aligned with international benchmarks. Continued evaluation, alongside piloting and phased implementation, will be essential to assess whether the revised audit items enhance the audit’s scope, drive service improvements and ultimately improve patient outcomes.

## Supplementary material

10.1136/bmjopen-2025-102789 online supplemental file 1

## Data Availability

Data are available upon reasonable request.
