# Supplementary material for: Enhancing acute stroke care in Ireland: A scoping review and Delphi consensus for the Irish National Audit of Stroke (INAS) dataset
Source: BMJ Open. 2025 Nov 21;15(11):e102789. doi: 10.1136/bmjopen-2025-102789 (PMC12658548; doi:10.1136/bmjopen-2025-102789)
Supplement: Supplementary file 2 [file bmjopen-2025-102789.reviewer_comments.pdf]

PEER REVIEW HISTORY

BMJ Open publishes all reviews undertaken for accepted manuscripts. Reviewers are asked to complete a checklist review form and are provided with free text boxes to elaborate on their assessment. These free text comments are reproduced below.

ARTICLE DETAILS

Title (Provisional)

Enhancing Acute Stroke Care in Ireland: A Scoping Review and Delphi Consensus for the Irish National Audit of Stroke (INAS) Dataset

Authors

Moran, Catherine Nora; Jeffares, Isabelle; McCormack, Joan; Merriman, Niamh A.; Bruen, Carlos; Jonsson, Agnes; Murphy, Paul; Rabbi, Khyber Afridi; Harbison, Joseph; Williams, David; Kelly, Peter; Collins, Rónán; Sexton, Eithne; Horgan, Frances; Ní Bheacáin, Máirín; Byrne, Elaine; Thornton, J; Tully, Collette; Hickey, Anne

---

VERSION 1 - REVIEW

**Reviewer**	**1**
**Name**	**Purvis, Tara**
**Affiliation**	**Monash University, Stroke and Ageing, School of Clinical Sciences at Monash Health**
**Date**	**28-May-2025**
**COI**	**None**

Thank you for this well written and valuable manuscript which outlines the process undertaken to develop an updated core dataset for integration into the Irish National Audit of Stroke (INAS), including a scoping review and consensus methods. While this is an important area of research for stroke quality improvement, but the process also has applicability more widely. However, there are several limitations that could be addressed.

One general comment is that the word audit and registry seem to have been used a little interchangeably throughout the manuscript. ‘National stroke audit’ has been defined within the *Methods concept.* However, the distinction between audit and registry is not clear, which I think is important for readers who may not be familiar with the area.

When was the INAS established? It seems quite advanced, so it is of interest what initiated the focus on updating the core dataset at this time? How were the core items decided upon on inception?

Abstract methods (line 132-133 p6)– 'Audit documentation was retrieved from identified audits……' are authors referring to data elements relating to the audits/registries? ?not just audit items as additional information about setting, and context were also collected?

Acknowledging words are limited within the abstract, however, authors mention '21 eligible international stroke audits and registries were identified', but there is no outline of the eligibility criteria.

Detailed information related to the Delphi rounds and consensus process is provided in Supplemental File 2. However, there is benefit in including some of these details in the abstract and main text for those who may not look at the supplements. For example, in the abstract – 'Stakeholder input to refine the dataset was gathered through a three-round Delphi consultation - which involved what…..? rating items using an anonymised review form and iterative discussion during stakeholder meetings? Also, as a reader, the process outlined in Supplemental File 2, which follows a more chronological flow, is easier to follow than how it is presented in the main text – authors could consider aligning. A figure may also help convey the overall process reported here, including the stages within the scoping review and Delphi consultation, and ensure consistent terminology is used throughout. The ACCORD guideline is also useful in identifying essential elements to report in consensus studies to enhance replication and consistency.

Can authors please describe how the qualitative responses were analysed.

On page 10 – phases 2 and 3 are used to reference future work related to rehab/recovery care and translation. However, phase 2 related to the current work is referred to within the supplemental files, which is a little confusing as a reader.

Interesting that authors chose to exclude RES-Q – while it is not specific to a country it could be considered one of the largest international stroke quality registries, and certain countries have good coverage with usage – I see the Thai Registry was initially included in this review (then excluded as not continuous data collection)– with 76 participating hospitals in 2008-2010, but currently, there appear to be 135 registered hospitals in Thailand using RES-Q (2024), so one might say it could potentially be the 'national country-wide system for stroke care data collection'. Author could consider a sentence in the discussion recognising the broad use of RES-Q and similarities/disparities?

Good to define all acronyms on first use – e.g. AuSCR defined on p 19, but used twice prior, also REC (p11) , ESO, RENISEN

Stakeholders are described on p16, and it is great to see that consumer representatives were included. It would be good to understand how many of the 24 panel members were involved with the INAS - ?as a user, but also overseeing content/implementation as mentioned.

Seems that some results are presented in the methods section – p16 – '…97 frequently collected international acute stroke care items' p17 –'….stakeholders reviewed 22 new items suggested in previous voting rounds…'

And conversely, potentially some methods are repeated in the results – e.g. line 453-456 and p459-462 p 21

Of interest, 4.9% of items had changed responses – it would be good to understand if this was towards inclusion or exclusion?

Good to include N for each rounds in Supplemental File 6/7

I may be misunderstanding, but for international items to get to the Delphi stage of item review, it was required that they were being used in at least 4 audits/registries, and that the AuSCR was the prime comparator for this step. If so, I am unclear as to why a single 'country of audit' is included in Supplemental File 7?

It is interesting that mRS at 90 days was included in the file dataset. How is this to be obtained? Does the INAS included PROMs collected after the acute admission?

The discussion includes some new data not presented in the results, particularly related to specifics around the additional items for inclusion/exclusion such as arrival at hospital, severity indicators and risk factors. Authors could consider briefly summarising some of these details in the results to ensure continuity with the discussion.

The conclusion is quite long and should not include references. Some of the points could be better placed in the discussion, particularly related to the next steps and future.

Reviewer **2**

Name **Adeel, Muhammad**

Affiliation **National Taipei University, International College of Sustainability Innovations**

Date **05-Jul-2025**

COI **None**

Thank you for the opportunity to review this impressive research titled "Development of a National Stroke Audit to Enhance the Quality of Acute Stroke Care in Ireland: A Scoping Review and Delphi Consensus Synthesis." The aims, methodology, and other aspects of this study are clearly articulated. I have only minor comments related to this manuscript.

Comments

1. On Page 12, Line 258, the reference pertains solely to research published in English. However, on Page 13, Line 276, it is stated that reports in languages other than English were included in the synthesis. Could you please clarify the reason for incorporating reports in non-English languages in the synthesis?

2. The statement references Page 14, Line 288, which mentions the inclusion of both acute and non-acute stroke reports. It requests clarification on the rationale behind including non-

acute stroke reports as part of the inclusion criteria, given that the criteria explicitly specify only acute stroke.

3. Page 27, Line 534, contains a typo in the mRS scale as modified Rankin scake. Please revise it.

4. Please explain the rationale for the search criteria from 2010 to 2021/2024. As mentioned in the Characteristics of Sources of Evidence, most registries began in the late 1990s and early to mid-2000s. Is it necessary to expand the search criteria from 2000 to the present?

**Reviewer**	**3**
**Name**	**Aprile, Irene**
**Affiliation**	**Fondazione Don Carlo Gnocchi Onlus**
**Date**	**09-Jul-2025**
**COI**	**None**

In the manuscript "Development of a National Stroke Audit to Enhance the Quality of Acute Stroke Care in Ireland: A Scoping Review and Delphi Consensus Synthesis", the Authors aim to develop, through a scoping review and Delphi consensus process, a core data for acute stroke care in Ireland to integrate into the national audit

The aim is interesting and could fit the Journal scope. However, i recommend major revisions before publication, primarily to improve clarity, specificity, and narrative flow. All critical points are included below:

Title

The current title is clear but slightly long. Consider a more concise version, for example "Enhancing Acute Stroke Care in Ireland: A Scoping Review and Delphi Consensus for a National Audit Dataset".

Abstract

The abstract could be made clearer in some sections: the methods are described in a very general way (without specifying the steps involved in the Delphi process and the scoping review), and the conclusion is rather vague. It would be helpful to explain more clearly the potential implications for research and clinical practice.

Introduction

The introduction would benefit from a more explicit reflection—grounded in the current literature—on what gaps remain and how this study aims to address them. Additionally, the rationale for combining a scoping review with a Delphi process should be better argued, highlighting the synergy between evidence and stakeholder consensus.

Materials and Methods

The Delphi methodology should be described in greater detail. Some information (e.g., the time frame considered) should be made easier to locate within the text. The handling of items in languages other than English should be clarified (Who carried out the translation? Using which tools or procedures?). Consider adding visual elements to improve the accessibility and readability of this section.

Results:

Consider stating the total number of included studies at the beginning of the section. Clarify what is meant by "commonly" and "infrequently" collected items, and specify the thresholds used to define frequency levels. The inclusion of visual elements would also enhance clarity and usability.

Discussion

Introduce direct comparisons with similar audits. Reconsider certain assertive phrases that emphasise the strengths of the study, such as "the present research offers a robust foundation for understanding acute stroke care and audit processes on an international scale."

Conclusions

Add new elements or a concise synthesis beyond what has already been discussed. Clarify future directions for the project and introduce a concrete operational proposal.

VERSION 1 - AUTHOR RESPONSE

Reviewer: 1
Ms. Tara Purvis, Monash University

Comments to the Author:
Thank you for this well written and valuable manuscript which outlines the process undertaken to develop an updated core dataset for integration into the Irish National Audit of Stroke (INAS), including a scoping review and consensus methods. While this is an important area of research for stroke quality improvement, but the process also has applicability more widely. However, there are several limitations that could be addressed
*Please see the attached report from this reviewer

Response:
We sincerely thank Ms. Purvis for the detailed, constructive, and insightful feedback on our manuscript. Below, we provide a point-by-point response to each comment, including a summary of changes made to the manuscript in response. We appreciate the reviewer's comments, which have helped improve the clarity, coherence, and transparency of our manuscript.

One general comment is that the word audit and registry seem to have been used a little interchangeably throughout the manuscript. 'National stroke audit' has been defined

within the *Methods concept.* However, the distinction between audit and registry is not clear, which I think is important for readers who may not be familiar with the area.

Response:

Thank you. We have clarified this in the Introduction. A registry refers to the structured collection of clinical data, while an audit uses that data to assess care quality and support improvement. As noted in the manuscript on page 9, these terms are often used interchangeably across the literature, especially when systems serve both functions.

Page 9: *"While the terms audit and registry are often used interchangeably, a registry primarily refers to the structured, longitudinal collection of patient-level clinical data, whereas an audit encompasses the systematic process of using that data to assess compliance with predefined standards, support benchmarking, and drive quality improvement (14, 15). In practice, this distinction often overlaps, particularly when national datasets serve both data collection and quality improvement functions."*

When was the INAS established? It seems quite advanced, so it is of interest what initiated the focus on updating the core dataset at this time? How were the core items decided upon on inception?

Response:

We thank the reviewer for this question. In response, we have expanded the relevant paragraph of the Introduction to provide further background on the establishment and development of the Irish National Audit of Stroke (INAS). The revised text on page 10 of the tracked manuscript now clarifies that INAS evolved from an earlier national stroke register, developed following the first Irish National Audit of Stroke Care in 2008, which identified critical gaps in stroke service delivery. The National Stroke Programme was established in 2010, and a stroke register was introduced . In 2019, governance of the register transferred to the National Office of Clinical Audit (NOCA), at which point the initiative was formalised as the Irish National Audit of Stroke (INAS). The updated paragraph reads:

Page 10: *"In Ireland, the first Irish National Audit of Stroke Care (2008) revealed significant shortcomings in the provision of stroke services across the hyper-acute, acute, and rehabilitation phases of care (20). In response, the National Stroke Programme (NSP) was established in 2010, leading to the development of a National Stroke Register to systematically collect data on stroke care delivered in public hospitals to target quality improvement initiatives (21). In 2019, governance of the Register was assumed by the National Office of Clinical Audit (NOCA), at which point the NSP was formalised and renamed as the Irish National Audit of Stroke (INAS) (22). INAS is a clinically led, continuous national clinical audit, overseen by a multidisciplinary Governance Committee comprising clinical experts, researchers, senior health system administrators, and public and patient representatives. This transition initiated a focus on updating the core dataset through a process of international benchmarking and stakeholder consensus."*

Abstract methods (line 132-133 p6)– 'Audit documentation was retrieved from identified audits......' are authors referring to data elements relating to the audits/registries? ?not just audit items as additional information about setting, and context were also collected?

Response:

Thank you for this observation. We have revised the Abstract section 'Data Extraction and Synthesis' to clarify that audit documentation refers not only to data items but also to supporting materials such as data dictionaries, item definitions, and contextual metadata. The updated sentence now reads:

Pages 6-7: *"Audit documentation (data dictionaries, item definitions, and contextual metadata) was retrieved from eligible audits. Acute stroke care items were extracted, charted and benchmarked against existing INAS items and each other to identify commonalities and gaps."*

Acknowledging words are limited within the abstract, however, authors mention '21 eligible international stroke audits and registries were identified', but there is no outline of the eligibility criteria.

Response:

Thank you for this comment. We have now added a brief summary of the eligibility criteria to the Abstract's 'Eligibility Criteria for Selecting Studies' section on page 6, as follows:

Page 6: *National stroke audits or registries with a country-wide scope, ≥1 year of continuous data collection, and active in 2021 were eligible. Only audits covering acute stroke care were included in this study phase. All records were screened for inclusion.*

The abstract has been reformatted and edited to fit journal guidelines and incorporate your recommended changes, while still meeting the 300 word limit.

Detailed information related to the Delphi rounds and consensus process is provided in Supplemental File 2. However, there is benefit in including some of these details in the abstract and main text for those who may not look at the supplements. For example, in the abstract – 'Stakeholder input to refine the dataset was gathered through a three-round Delphi consultation - which involved what.....? rating items using an anonymised review form and iterative discussion during stakeholder meetings? Also, as a reader, the process outlined in Supplemental File 2, which follows a more chronological flow, is easier to follow than how it is presented in the main text – authors could consider aligning. A figure may also help convey the overall process reported here, including the stages within the scoping review and Delphi consultation, and ensure consistent terminology is used throughout. The ACCORD guideline is also useful in identifying essential elements to report in consensus studies to enhance replication and consistency.

Response:

We thank the reviewer for this constructive feedback. In response, we have revised the "Stakeholder Delphi Consultation" section in the main Methods on **pages 18-20 of** the tracked manuscript to better align with the chronological structure used in Supplemental File 2. The revised text now clearly describes the three Delphi rounds, the voting methods, consensus thresholds, and the use of iterative discussion to inform stakeholder decisions. We have ensured consistency in terminology throughout the manuscript and clarified how items were presented and assessed.

We have also added a sentence to the Abstract to briefly describe how stakeholder input was gathered:

Pages 6-7: *"A three-round Delphi process with 24 national stakeholders (clinicians, nurses, allied health professionals, researchers, policymakers, and patient representatives) was conducted to audit and refine the dataset through structured, anonymised item rating, iterative feedback, and consensus-building discussions."*

We also now cite the ACCORD guidelines in the Methods section.

Page 12: "This study is also reported in accordance with the ACCORD (ACcurate COnsensus Reporting Document) Guideline (36)."

Can authors please describe how the qualitative responses were analysed.

Response:

During the Delphi process, participants were invited to provide optional qualitative comments alongside their voting responses. These brief, item-specific comments were reviewed and summarised by the study team after each round. The anonymised summaries were presented at Steering Group and Governance Committee meetings to support discussion and contextualise group voting trends. In addition, transcripts of the stakeholder meetings were shared with all participants following each round, allowing them to review group-level commentary and reflections prior to the next stage of voting. This approach facilitated iterative feedback and transparency, although the qualitative data were not formally analysed using a structured coding framework, as the primary aim was to support consensus development. We have added the following clarification to the Methods on page 19 of the tracked manuscript section:

Page 19: *"Each item was rated as "Include in INAS," "Exclude from INAS," "No strong view," or "Don't know enough to be able to say," with space provided for brief optional qualitative comments. These comments were anonymised and summarised by the research team to contextualise voting patterns and inform discussion. Summarised feedback was presented during Steering Group and Governance Committee meetings between voting rounds, and meeting transcripts containing aggregated, anonymised comments were circulated to all participants. This process promoted transparency, allowed participants to reflect on group perspectives, and informed subsequent voting. In round 2 of the Delphi process, participants had the opportunity to revise their initial responses in light of this comparative feedback and group-level voting patterns. Although qualitative comments were not formally coded, they were instrumental in guiding iterative consensus-building throughout the process. Based on*

stakeholder engagement, additional items were proposed during this stage and incorporated into the inventory for consideration in the final Delphi round.

In the final round of the Delphi process (Round 3), participants voted on a reduced set of items based on the outcome of the first two rounds, as well as the additional expert-suggested items, using simplified response options: "Include in INAS," "Exclude from INAS," or "Don't know enough to be able to say." Anonymised summaries of prior discussions were provided to guide decisions. Final consensus levels were reviewed by the Steering Group and Governance Committee, which provided clinical and policy justification for the resulting core minimum dataset."

We have also added in the following section to the Delphi description in **Supplemental File 2:**

"The percentage breakdown of stakeholder responses and a summary of anonymised qualitative feedback were aggregated and presented at Steering Group and INAS Governance Committee meetings. Descriptive Round 1 results were circulated to participants in tabular form. Qualitative comments submitted alongside voting responses were anonymised and summarised by the research team to contextualise voting patterns and inform discussion. Meeting transcripts and feedback summaries were shared with all participants to promote transparency and support reflection ahead of subsequent rounds. While not formally coded, this input was instrumental in guiding the iterative consensus-building process."

On page 10 – phases 2 and 3 are used to reference future work related to rehab/recovery care and translation. However, phase 2 related to the current work is referred to within the supplemental files, which is a little confusing as a reader.

Response:

Thank you for flagging this source of confusion. In the main manuscript, "Phase 2" and "Phase 3" on page 11 (Introduction) refer to later programme work (rehabilitation/recovery and implementation/translation) beyond the acute-care focus of this paper.

Page 11: *"The aim of this research was to develop INAS by establishing a core minimum dataset for acute care (Phase 1), starting with a scoping review of international stroke audit, followed by an international benchmarking exercise of stroke audit items, and iterative cycles of expert stakeholder engagement in order to agree a final acute audit dataset. The development of the audit dataset for rehabilitation and recovery care (Phase 2) and the identification of resourcing needs and production of an implementation strategy (Phase 3) are reported separately (27)."*

In Supplemental materials we had inadvertently used similar "Phase" language to describe steps in the acute international benchmarking workflow, which was misleading. We have replaced the word "phase" in the supplemental files to "Step 1" and "Step 2"

(benchmarking steps), and have updated the methods section in the manuscript to also use the word "step" when describing the acute benchmarking process.

e.g. **Page 16:** *"Once the data items were collated, a two-step benchmarking exercise was conducted … In the first step"*

Interesting that authors chose to exclude RES-Q – while it is not specific to a country it could be considered one of the largest international stroke quality registries, and certain countries have good coverage with usage – I see the Thai Registry was initially included in this review (then excluded as not continuous data collection)– with 76 participating hospitals in 2008- 2010, but currently, there appear to be 135 registered hospitals in Thailand using RES-Q (2024), so one might say it could potentially be the 'national country-wide system for stroke care data collection'. Author could consider a sentence in the discussion recognising the broad use of RES-Q and similarities/disparities?

Response:

Thank you. We have now acknowledged RES-Q's broad international role and detailed its overlap with both current and proposed INAS items. This has been added to the Discussion on Page 33 in the tracked manuscript section to clarify its relevance.

Page 33: *"Although RES-Q was excluded from our main dataset comparison, we acknowledge its broad international use and relevance to stroke quality improvement. Notably, there is substantial overlap between RES-Q items (84) and both current and proposed INAS items. For example, RES-Q includes "mode of arrival to hospital," which aligns with the newly proposed INAS item on whether the patient arrived by ambulance. It also captures NIHSS on arrival and a range of pre-stroke risk factors, which mirror several of our proposed INAS item additions. RES-Q similarly collects data on pre-admission medications (e.g. antihypertensives, lipid-lowering agents), as well as key post-stroke complications including pulmonary embolism (PE), urinary tract infections (UTIs), and deep vein thrombosis (DVT), all of which are now recommended for inclusion in INAS. This alignment underscores the validity of our proposed additions and suggests that future efforts to harmonise INAS with international standards may be highly beneficial, particularly in jurisdictions where RES-Q is widely used."*

Good to define all acronyms on first use – e.g. AuSCR defined on p 19, but used twice prior, also REC (p11) , ESO, RENISEN

Response:

Thank you for spotting this. We have reviewed the manuscript to ensure all acronyms are now defined at first use. Specifically, OSF, REC, RES-Q, and AuSCR have been defined in the Materials and Methods section; RENISEN and Riksstroke in the Results section; and ESO and NIHSS in the Discussion. We have also checked for consistency throughout the text.

Stakeholders are described on p16, and it is great to see that consumer representatives were included. It would be good to understand how many of the 24 panel members were involved with the INAS - ?as a user, but also overseeing content/implementation as mentioned.

Response:

Thank you for this suggestion. We have expanded the description of stakeholder involvement in the Methods section on page 18 of the tracked manuscript to clarify how many of the 24 panel members had direct experience with the INAS.

Page 18: *"A majority of stakeholders (n=18) were very familiar with INAS and almost all healthcare professional stakeholders were involved in inputting or overseeing the input of data from their hospital to the INAS database. Stakeholders included the National Lead of the Stroke Programme in Ireland, the Clinical Lead of INAS and the INAS Programme Manager. This inclusive selection ensured broad representation of relevant national stakeholders, particularly those who oversee INAS audit content and implementation, to enhance the validity and applicability of the audit's findings".*

Seems that some results are presented in the methods section – p16 – '...97 frequently collected international acute stroke care items' p17 –'....stakeholders reviewed 22 new items suggested in previous voting rounds...'

Response:

Thank you for highlighting this. We have removed specific item counts from the Methods. Instead, we now describe the dataset more generally. Specific figures (e.g., 97 international items and 22 expert-suggested items) are now reserved for the Results section. We have revised the relevant text on pages 18-19 in the Methods section to read:

Page 18: *"Stakeholders with expertise in stroke care were invited to participate in a multi-round Delphi consultation aimed at evaluating and refining existing INAS items, as well as frequently-collected international items (used in four or more national audits or registries)."*

Page 19: *"Based on stakeholder engagement, additional items were proposed during this stage and incorporated into the inventory for consideration in the final Delphi round."*

And conversely, potentially some methods are repeated in the results – e.g. line 453-456 and p459-462 p 21

Response:

Thank you for flagging this. We have revised these sections to focus on presenting the findings without repeating methodological detail.

Page 23: *"Comparison of INAS items with the international inventory showed varying levels of overlap: of the 103 INAS items, 40 were classified as highly shared, 48 as moderately shared, and 15 as low shared or not collected elsewhere (see Supplemental File 4 for full frequency data). This benchmarking exercise highlighted both areas of alignment with international practice and opportunities for dataset enhancement. Analysis of the international audit inventory identified 97 acute stroke care items not currently included in INAS but frequently collected elsewhere (these items appeared in at least four international stroke audits or registries (see Supplemental File 5)). Of these, 90 were used by a moderate number of audits, while 7 were commonly collected across a high number of registries. This subset of frequently collected international items was brought forward for stakeholder review to inform potential expansion of the INAS dataset."*

Of interest, 4.9% of items had changed responses – it would be good to understand if this was towards inclusion or exclusion? Good to include N for each rounds in Supplemental File 6/7

Response:

Thank you for this observation. In response, we have expanded our reporting of item-level voting changes to provide greater clarity. Specifically, we have:

- Disaggregated the response changes by dataset (INAS vs. international items), and by type of change: (1) votes changed from one eligible response to another, and (2) new responses provided in Round 2 for items that were left unanswered in Round 1.
- Summarised the direction of change (i.e., toward "Include," "Exclude," "No strong view," or "Don't know") in the Results section of the manuscript under Delphi Round 2.
- Added a new table (now Supplemental File 8) that outlines individual participant-level changes across both datasets, accompanied by an explanatory note to aid interpretation.
- Updated Supplemental Files 6 and 7 to include participant numbers at the top of each table and a more detailed breakdown of the sample composition in each round.
- Additionally, we have revised the accompanying table notes to more explicitly clarify the number of participants included in each round to support reader understanding.

We have clarified in the manuscript that:

Page 24: *"Across all stakeholder responses, a total of 66 INAS item responses (3.2% of all possible INAS responses, based on 103 items) and 42 international item responses (2.16% of all possible international responses, based on 97 items) were revised between Rounds 1 and 2 (see Supplemental File 8 for details). Of the 66 INAS item changes, 44 (66.7%) shifted toward "Include," 15 (22.7%) toward "Exclude," and 7 (10.6%) toward "No strong view." For the 42 international item changes, 10 (23.8%) shifted toward "Include," 17 (40.5%) toward "Exclude," 5 (11.9%) toward "Don't know," and 10 (23.8%) toward "No strong view." Vote distributions in Supplemental Files 6 and 7 represent combined responses from all 20 stakeholders, including unchanged votes from eight non-responders and two who confirmed their initial responses, along with updates from nine participants who submitted revisions."*

I may be misunderstanding, but for international items to get to the Delphi stage of item review, it was required that they were being used in at least 4 audits/registries, and that the AuSCR was the prime comparator for this step. If so, I am unclear as to why a single 'country of audit' is included in Supplemental File 7?

Response:

You are correct that the AuSCR was used as the primary comparator in the benchmarking process. However, once this initial comparison between INAS and AuSCR against all other international audits was completed, we extended the analysis to ensure comprehensiveness. Specifically, we identified additional items present in other international audits that were not included in either INAS or AuSCR. These remaining items were then benchmarked against the full set of included audits. Accordingly, in Supplemental File 7, the 'country of audit' refers to the source audit in which a given item was initially identified. This provides transparency about the origin of each item before its broader comparison across registries for inclusion consideration. However, to improve clarity we have made the following changes:

Methods Page 17: *"Following this primary benchmarking, a review of all remaining international audits was conducted to identify any additional acute care items not present in either INAS or AuSCR. These items were also cross-checked across the full audit set to determine their frequency of use. For transparency, the country of the audit where each item was first identified is noted as its source of origin."*

As a note under the tables in **Supplemental Files 5 and 7:** *"The "Country of Audit" refers to the audit or registry in which each item was first identified before being cross-checked against all other included audits to assess how frequently it was collected internationally."*

It is interesting that mRS at 90 days was included in the file dataset. How is this to be obtained? Does the INAS included PROMs collected after the acute admission?

Response:

While most INAS indicators relate to the acute phase, the mRS at 90 days was included due to its value in assessing long-term outcomes and its alignment with international standards. Though INAS does not currently collect post-discharge data, this measure is often recorded during routine outpatient follow-up at three months in Ireland, making its inclusion feasible. We have clarified this in the discussion.

Page 32: *"While most of these indicators are already within the remit of acute stroke audits some, like the mRS at 90 days, typically fall outside the immediate hospital admission period. However, this outcome measure is commonly assessed during standard outpatient follow-up visits at three months post-discharge in Ireland, providing a practical opportunity for its inclusion without significant additional data burden. Consequently, the implementation of these indicators will enable systematic and comparative assessments of stroke care quality across international healthcare systems."*

The discussion includes some new data not presented in the results, particularly related to specifics around the additional items for inclusion/exclusion such as arrival at hospital, severity indicators and risk factors. Authors could consider briefly summarising some of these details in the results to ensure continuity with the discussion.

Response:

Thank you for this observation. The items recommended for the core dataset are presented in Table 1 of the Results section, with newly proposed additions now marked by a bold asterisk. The number of items ultimately recommended for inclusion or removal is detailed in the text. In the Discussion, we have reduced the word count, and removed some text, and focused on summarising and reviewing the potential usefulness of the suggested changes, grouping recommendations rather than discussing individual items in detail.

The conclusion is quite long and should not include references. Some of the points could be better placed in the discussion, particularly related to the next steps and future.

Response:

Thank you for this suggestion. We have shortened the Conclusion and removed the reference, moving points detailing next steps and future directions to the Discussion section. The conclusion now states:

Page 35: *"This study aimed to develop a core outcomes dataset for acute stroke care in Ireland through international benchmarking and stakeholder consensus. It provides a structured overview of global audit practices and contributes a refined, context-specific dataset aligned with international benchmarks. Continued evaluation, alongside piloting and phased implementation, will be essential to assess whether the revised audit items enhance the audit's scope, drive service improvements, and ultimately improve patient outcomes."*

Reviewer: 2
Dr. Muhammad Adeel, National Taipei University

Comments to the Author:

Thank you for the opportunity to review this impressive research titled "Development of a National Stroke Audit to Enhance the Quality of Acute Stroke Care in Ireland: A Scoping Review and Delphi Consensus Synthesis." The aims, methodology, and other aspects of this study are clearly articulated. I have only minor comments related to this manuscript.

Response:

We sincerely thank Dr. Adeel for the positive and encouraging comments, and for highlighting areas for clarification and improvement. Below we provide point-by-point responses to each of your comments and detail revisions made to the manuscript in

response. We greatly appreciate the reviewer's thoughtful comments, which have helped improve the clarity and consistency of the manuscript.

Comments

1. On Page 12, Line 258, the reference pertains solely to research published in English. However, on Page 13, Line 276, it is stated that reports in languages other than English were included in the synthesis. Could you please clarify the reason for incorporating reports in non-English languages in the synthesis?

Response:

We thank the reviewer for highlighting this point. To clarify, the restriction to English-language publications applied solely to the peer-reviewed database searches (e.g., Medline, Embase, CINAHL), due to indexing constraints and the practical challenges of screening non-English academic literature.

However, for audits included in the review, we sought associated audit documentation, such as data dictionaries, metadata, and methodological guides, via audit websites or direct correspondence with audit leads. Where such documentation was available only in languages other than English, it was translated to enable benchmarking of data items across audits. Five non-English audit documents were translated and incorporated into the data synthesis. We have now clarified this in the "Methods: Information Sources" section on page 14 of the tracked manuscript with the following:

Page 14: *"Peer-reviewed literature searches were limited to English-language publications. However, for audits meeting inclusion criteria, supplementary audit documentation (e.g., data dictionaries, methodology reports) obtained via audit/registry websites or through direct correspondence with audit/registry leads was included regardless of language and translated to English where necessary to enable data benchmarking."*

2. The statement references Page 14, Line 288, which mentions the inclusion of both acute and non-acute stroke reports. It requests clarification on the rationale behind including non-acute stroke reports as part of the inclusion criteria, given that the criteria explicitly specify only acute stroke.

Response:

Thank you for this observation. We have now clarified the rationale for this inclusion. While the primary focus of this manuscript (Phase 1 of the study) is acute stroke care, some national audits and registries collect data spanning the full stroke care pathway, including rehabilitation and long-term follow-up. We confirm that, for the purposes of this paper reporting phase 1 of the study, only acute stroke care data items were extracted, analysed, and benchmarked. Data relating to rehabilitation and post-acute care from these broader audits are being addressed separately in Phase 2 of the project (to be reported elsewhere). Both phases shared a common initial scoping review search, but the data syntheses differed

according to the distinct scope of each phase. To clarify this in the manuscript, we have revised the relevant sentence on page 15 of the tracked manuscript to read:

Page 15: *"Audits that encompassed both acute and non-acute stroke care were eligible, provided they included a continuous and structured acute care component. During the data charting and synthesis steps, the included audit documents were categorised as relating to acute stroke care (Phase 1) or non-acute stroke care (Phase 2; this phase is reported separately). For the phase of the review reported here, only data items related to acute stroke care were extracted and analysed."*

3. Page 27, Line 534, contains a typo in the mRS scale as modified Rankin scake. Please revise it.

Response:

Thank you for identifying this error. It has been amended to "modified Rankin Scale."

4. Please explain the rationale for the search criteria from 2010 to 2021/2024. As mentioned in the Characteristics of Sources of Evidence, most registries began in the late 1990s and early to mid-2000s. Is it necessary to expand the search criteria from 2000 to the present?

Response:

Thank you for your comment. We selected 2010 as the lower limit for our search criteria to ensure that the audit documentation reflected contemporary clinical practice, dataset structures, and ongoing audit operations. While we acknowledge that many national stroke audits were established prior to 2010, our primary objective was to capture documentation from audits that were still active and relevant to current benchmarking. The 2010 threshold also supported feasibility by managing the scope and volume of data retrieved. Importantly, because one of our inclusion criteria was that audits had to be continuously active as of 2021 (the time of the initial search), audits initiated prior to 2010 would still have been captured if they remained operational. We have added the following line to the Eligibility Criteria section:

Pages 12-13: *"We limited inclusion to sources from 2010 onward to ensure the data reflected contemporary clinical practice and actively maintained audits relevant to current benchmarking."*

Reviewer: 3
Dr. Irene Aprile, Fondazione Don Carlo Gnocchi Onlus

Comments to the Author:

In the manuscript "Development of a National Stroke Audit to Enhance the Quality of Acute Stroke Care in Ireland: A Scoping Review and Delphi Consensus Synthesis", the Authors aim

to develop, through a scoping review and Delphi consensus process, a core data for acute stroke care in Ireland to integrate into the national audit

The aim is interesting and could fit the Journal scope. However, i recommend major revisions before publication, primarily to improve clarity, specificity, and narrative flow. All critical points are included below:

Response:

We sincerely thank the reviewer for the thoughtful and constructive comments that have helped us significantly improve the manuscript's clarity, structure, and impact. We address each comment below and indicate how the manuscript has been revised in response.

Title: **The current title is clear but slightly long. Consider a more concise version, for example "Enhancing Acute Stroke Care in Ireland: A Scoping Review and Delphi Consensus for a National Audit Dataset".**

Response:

Thank you for this helpful suggestion. We have revised the title in line with your recommendation and to align the wording with INAS (Irish National Audit of Stroke):

"Enhancing Acute Stroke Care in Ireland: A Scoping Review and Delphi Consensus for the Irish National Audit of Stroke (INAS) Dataset."

Abstract

The abstract could be made clearer in some sections: the methods are described in a very general way (without specifying the steps involved in the Delphi process and the scoping review), and the conclusion is rather vague. It would be helpful to explain more clearly the potential implications for research and clinical practice.

Response:

Thank you for this feedback. We have revised the abstract to more clearly describe the steps involved in both the scoping review and the Delphi process, and have expanded the conclusion to highlight potential implications for research and clinical practice. In addition, the abstract has been restructured according to the journal's required headings to improve clarity and accessibility of the study methodology.

Introduction

The introduction would benefit from a more explicit reflection—grounded in the current literature—on what gaps remain and how this study aims to address them. Additionally, the rationale for combining a scoping review with a Delphi process should be better argued, highlighting the synergy between evidence and stakeholder consensus.

Response:
Thank you for this suggestion. We have expanded the Introduction (page 11 of the tracked manuscript) to more explicitly outline existing gaps, grounded in the current literature, and how this study aimed to address them. We have also added more context to the rationale for combining a scoping review with a Delphi process.

Page 11: *"Despite the availability of stroke audits and registers internationally, a number of challenges prevail. Many countries do not have a national stroke registry or audit, with only 14% of WHO member countries and territories having what is described as optimal stroke surveillance through a national stroke registry (28). Where such national registries or audits are available, there are frequently limitations that pose challenges to data interpretation and application. These include lack of consistency in definitions, in the methods used to gather data, errors in data entry with no data validation checks built into the data collection process, lack of precision in coding of stroke, and lack of international benchmarking (27-29)."*

Page 11: *"This approach of combining a scoping review with a Delphi process enabled us to ensure that the process was well-informed and evidence-based, enabling the stakeholders involved in the Delphi process to make decisions about the items suitable for inclusion in and exclusion from INAS based on best current evidence of international stroke audit."*

Materials and Methods

The Delphi methodology should be described in greater detail. Some information (e.g., the time frame considered) should be made easier to locate within the text.

Response:

We thank the reviewer for this suggestion. In response, we have expanded the description of the Delphi process in the main manuscript (see **pages 18-20** of the tracked manuscript). The section now includes additional detail on consensus thresholds, timing (March–December 2022), the item selection and revision process across rounds, and how discussion meetings were integrated into the iterative feedback cycle. We have also clarified that voting thresholds and procedures were pre-specified, and that anonymised group results and stakeholder discussions informed decisions in later rounds. While the Supplemental File still contains detailed round-specific instructions and methodology, the Methods section in the main manuscript now provides sufficient detail for readers to understand the Delphi approach.

The handling of items in languages other than English should be clarified (Who carried out the translation? Using which tools or procedures?).

Response:

We thank the reviewer for clarification request. We have now specified in the manuscript that translations of non-English audit documentation were carried out by a professional translation service. Specifically, documents were translated by Certified Translations

(www.certifiedtranslations.ie), a Dublin-based certified translation provider. All translations were completed by professional translators and verified for accuracy. We have updated the relevant section in the Methods- Search Strategy and Selection Criteria on Page 14 to reflect this.

Page 14: *"Non-English audit documents were translated into English using a professional certified translation service (Certified Translations, Dublin, Ireland (www.certifiedtranslations.ie)) to ensure data accuracy and completeness during synthesis."*

Consider adding visual elements to improve the accessibility and readability of this section.

Response:

Thank you for this suggestion. We have enhanced the accessibility and readability of this section by adding further clarifying detail, reorganising the content with descriptive subheadings, and refining the structure to improve the logical flow.

Results: Consider stating the total number of included studies at the beginning of the section.

Response:

Thank you for this suggestion. Because our unit of inclusion was the audit or registry rather than individual articles, we have not reported a total number of included articles. Relevant information was extracted from any source referencing an eligible audit, and multiple sources could refer to the same audit or registry. Reporting the number of articles would therefore be misleading in the context of our methodology. We have clarified this in the Results section and in the note beneath the PRISMA flow diagram in Figure 1.

Results page 21: As the unit of inclusion was the audit or registry rather than individual articles, multiple sources could refer to the same audit or registry.

Figure note: *"Note. Inclusion was at the audit/registry level; multiple sources could inform a single audit, so article counts are not reported."*

Clarify what is meant by "commonly" and "infrequently" collected items, and specify the thresholds used to define frequency levels. The inclusion of visual elements would also enhance clarity and usability.

Response:

Thank you for this comment. We have clarified the thresholds for how commonly an item is collected internationally within the Methods section, supplementing the information already provided in the supplemental table legends. Specifically, we now state:

Page 17: *"An INAS item was classified as low shared if present in 0–2 other audits/registries, moderately shared if present in 3–10, and highly shared if present in 11–20."*

Page 17: *"An international reference item not collected by INAS was classified as moderately shared if present in 3–10 other audits/registries, and highly shared if present in 11–19."*

We also note that only frequently collected international items were put forward for discussion in the Delphi process, as already defined within the Methods:

Page 17: *"Following consultation with the project Steering Group, a criterion was set such that only predominant frequently collected international items, that is, those items shared amongst four or more audits/registries in total (i.e., the comparator audit item and at least 3 other audits/registries), were put forward for further consideration by a stakeholder panel."*

In line with another reviewer's feedback, these details remain in the Methods rather than being repeated in the Results, where we instead use the classification terms (low, moderate, high sharing) for clarity and brevity.

Discussion

Introduce direct comparisons with similar audits. Reconsider certain assertive phrases that emphasise the strengths of the study, such as "the present research offers a robust foundation for understanding acute stroke care and audit processes on an international scale."

Response:

Thank you for this suggestion. The international benchmarking exercise conducted as part of this study serves as a structured comparison between the INAS dataset and 20 international audits/registries. Frequency counts illustrating the collection of each item across audits are included in Supplemental Files 4 and 5, providing a detailed comparative overview that guided item selection.

However, we have now addressed this point in the revised manuscript by incorporating a direct comparison between the proposed INAS dataset and the RES-Q registry within the Discussion section. Specifically, we note areas of overlap with existing INAS items, as well as similarities between our newly proposed additions and RES-Q data elements (e.g., arrival mode, NIHSS, pre-stroke risk factors, medication use, and post-stroke complications).

Page 33: "*Although RES-Q was excluded from our main dataset comparison, we acknowledge its broad international use and relevance to stroke quality improvement. Notably, there is substantial overlap between RES-Q items (84) and both current and proposed INAS items. For example, RES-Q includes "mode of arrival to hospital," which aligns with the newly proposed INAS item on whether the patient arrived by ambulance. It also captures NIHSS on arrival and a range of pre-stroke risk factors, which mirror several of our proposed INAS item additions. RES-Q similarly collects data on pre-admission medications (e.g. antihypertensives, lipid-lowering agents), as well as key post-stroke complications*

including pulmonary embolism (PE), urinary tract infections (UTIs), and deep vein thrombosis (DVT), all of which are now recommended for inclusion in INAS. This alignment underscores the validity of our proposed additions and suggests that future efforts to harmonise INAS with international standards may be highly beneficial, particularly in jurisdictions where RES-Q is widely used."

We have also reviewed the phrasing and have revised some sentences to temper the tone while still acknowledging the contribution of the work. We have also removed the word ideal and made small changes to the text. The revised sentences now read:

Page 34: *"The scoping review and Delphi consensus process described here represents a structured approach to developing a core outcomes dataset for acute stroke care in Ireland, grounded in international benchmarking and broad stakeholder input. The findings highlight the importance of periodically reviewing audit items to maintain alignment with evolving guidelines and clinical practice, and to refine item clarity through rewording, expanded response options, and improved sequencing with skip logic. Implementation of the revised dataset is now underway, led by the INAS Governance Committee in collaboration with the multi-stakeholder project Steering Group, with careful attention to the resourcing implications of these changes. Piloting the dataset will provide valuable insights into its feasibility and potential impact, supporting evidence-informed adjustments prior to full integration. This applied partnership approach illustrates how research can be translated rapidly into actionable audit policy and practice, ultimately strengthening the capacity for continuous improvement in stroke care."*

Conclusions

Add new elements or a concise synthesis beyond what has already been discussed. Clarify future directions for the project and introduce a concrete operational proposal.

Response:

Thank you for this suggestion. We have incorporated the study's implications and concrete operational steps into the Discussion section (see text quoted in our previous response to the reviewer) and revised the Conclusion to be more concise, focusing on key take-home messages.

Page 35 Conclusions: *"This study aimed to develop a core outcomes dataset for acute stroke care in Ireland through international benchmarking and stakeholder consensus. It provides a structured overview of global audit practices and contributes a refined, context-specific dataset aligned with international benchmarks. Continued evaluation, alongside piloting and phased implementation, will be essential to assess whether the revised audit items enhance the audit's scope, drive service improvements, and ultimately improve patient outcomes."*

Reviewer: 1
If you have selected 'Yes' above, please provide details of any competing interests.: Not applicable

Reviewer: 2
If you have selected ‘Yes’ above, please provide details of any competing interests.: Not applicable

Reviewer: 3
If you have selected ‘Yes’ above, please provide details of any competing interests.: Not applicable

---

VERSION 2 - REVIEW

Reviewer **1**

Name **Purvis, Tara**

Affiliation **Monash University, Stroke and Ageing, School of Clinical Sciences at Monash Health**

Date **03-Oct-2025**

COI

Thank you for considering and incorporating the feedback. The manuscript is much improved, and I have no further recommendations.
